# Porous phosphate-based bioactive glass /β-TCP scaffold for tooth remineralization

**DOI:** 10.1371/journal.pone.0284885

**Published:** 2023-05-18

**Authors:** Criseida Ruiz-Aguilar

**Affiliations:** Escuela Nacional de Estudios Superiores Unidad Juriquilla, Universidad Nacional Autónoma de México, Juriquilla, Queretaro, México; Saveetha Institute of Medical and Technical Sciences: Saveetha University, INDIA

## Abstract

The total or partial loss of teeth in the Mexican population due to periodontal diseases and trauma causes the development of other conditions, such as limitations in chewing and grinding food, pronunciation difficulties, and oral aesthetic alterations. In Mexico, oral diseases have been described to affect 87% of the population, according to reports by the health services, emphasizing that pregnant women and patients with diabetes mellitus have the highest risk of presenting with severe periodontal diseases or tooth loss, according to findings by the Mexican Health Department’s Specific Action Program for the prevention, detection, and control of oral health problems (2013–2018). There was a 92.6% prevalence of dental caries in the population examined, and the prevalence of periodontal problems, mainly in 40-year-olds, was above 95%. The objective of this investigation was to manufacture and characterize porous 3D scaffolds with innovative chemical compositions, using phosphate-based bioactive glass, beta-phase tricalcium phosphate, and zirconium oxide, in variable quantities. The scaffold manufacturing method combined two techniques: powder metallurgy and polymer foaming. The results obtained in this research were promising since the mechanically tested scaffolds showed values of compressive strength and modulus of elasticity in the range of human trabecular bone. On the other hand, the *in vitro* evaluation of the samples immersed in artificial saliva at days 7 and 14 presented the calcium/phosphorus ratio = 1.6; this value is identical to the reported state-of-the-art figure, corresponding to the mineral phase of the bones and teeth. Likewise, the precipitation of the flower-like morphology was observed on the entire surface of the scaffold without zirconia; this morphology is characteristic of hydroxyapatite. On the other hand, the samples with 0.5 and 1.0 mol% zirconia showed less hydroxyapatite formation, with a direct correlation between scaffold dissolution and the amount of zirconia added.

## 1. Introduction

According to the data reported in 2019 by the Mexican Institute of Social Security (*IMSS*), 78% of Mexicans presented with caries problems, and 60% of the population manifested periodontal diseases [1].

The loss of calcium and phosphorus produces softening in the hard tissues, both in bones and in the dental layers, directly affecting the enamel, dentin, the nerves of the blood vessels, and the non-calcified connective tissue. These conditions promote the formation of cavities, mouth functionality limitations, and in difficult situations, the loss of teeth, altering the dental and facial aesthetics of the individual [2]. If the demineralization process and the progressive loss of hydroxyapatite (HA) mineral ions that make up the enamel, dentin, and tooth [3] are added to the abovementioned conditions, they cause a complex situation for patient tooth recovery.

On the other hand, porous scaffolds are a successful alternative for various applications in bone tissue engineering. These 3D templates make it possible to functionalize new tissues and repair and regenerate structures in the human body [4]. There are various techniques for manufacturing 3D porous scaffolds, and rapid prototyping highlights powder metallurgy, gas foaming, electrospinning, stereolithography, and bioprinting, among others [5]. Each scaffold manufacturing technique provides physical, chemical, mechanical, and structural properties for trabecular and cortical bone tissue engineering applications.

Furthermore, calcium phosphate ceramics and bioactive glasses have a chemical composition similar to that of bones and teeth [6–8]. They show high osteoconductivity, osteoinductivity [9], injectability [10], resorbability [11], crystallinity [12], and biocompatibility [13]. These properties make them excellent candidates for use in the regeneration and healing of dental structures [14–17].

Some of the most widely used applications of bioactive glasses and calcium phosphates are in craniomaxillofacial reconstruction, fixation systems, bone fillings, periodontal regeneration, and maxillary sinus floor elevation, among others [18, 19].

The present study developed a new chemical formulation of phosphate-based bioactive glass, β-phase tricalcium phosphate (β-TCP), and zirconia (ZrO_2_), in variable amounts. These are used in manufacturing 3D porous scaffolds, which improve porous scaffold resorption, in contact with artificial saliva, for dental remineralization treatment.

## 2. Methodology

The methodology for preparing the foams was in 3 stages described below.

### 2.1 Manufacture of phosphate-based glass

In the phosphate-based bioactive glass preparation, the chemical composition was in molar quantities of 45 P_2_O_5_−30 CaO–(25-X) Na_2_O, where X = 0, 0.5, and 1.0 of ZrO_2_. Phosphorus pentoxide (P_2_O_5_) dehydrated dibasic calcium phosphate (CaHPO_4_.2H_2_O) and monobasic sodium phosphate (NaH_2_PO_4_) were the powder chemical reagents, with a purity ≥98% (Sigma-Aldrich). The chemical precursors were mixed in a conventional ball mill for 30 minutes after melting and quenching. The thermal cycle was carried out in a Carbolite HTF 1700 oven, with a heating rate of 10°C/min until reaching 400°C for a residence time of 90 min. The temperature was then raised to 1100°C for 90 min, and the melt was quenched on a stainless-steel plate in air at room temperature.

### 2.2 Manufacture of β-phase tricalcium phosphate

β-TCP was synthesized using the mechano-synthesis route in a Restch PM400 high-energy planetary mill for 12 h at 350 revolutions per minute (rpm), with a ball/mass (g) ratio of 8:1; the stainless-steel balls measured 10 mm in diameter. The precursors used were reagent-grade powders of calcium carbonate (CaCO_3_) and dibasic calcium phosphate (CaHPO_4_) from Sigma-Aldrich. The precursors were mixed in a conventional ball mill, maintaining the calcium/phosphorus ratio, according to the reaction indicated in Eq (1):

$$2{\text{CaHPO}}_{4}.2{\text{H}}_{2}\text{O}+{\text{CaCO}}_{3}\to {\text{Ca}}_{3}{\left({\text{PO}}_{4}\right)}_{2}+5{\text{H}}_{2}\text{O}+{\text{CO}}_{2}$$ (1)

After the homogenization of the precursors, mechano-synthesis was carried out. Later, heat treatment was performed at 900°C with a heating rate of 10°C/min for 3 h in a Thermolyne Benchtop muffle furnace, model FB-1315M. The formation of the β phase was confirmed using X-ray diffraction and JCPDS card No: 01-073-4869.

### 2.3 Manufacture of scaffold

The 3D porous scaffolds were manufactured with a mixture of 55% wt powders of tricalcium phosphate and phosphate-based bioactive glass (ratio β-TCP/bioactive glass = 80/20% wt). 2.5% wt of p-toluene sulfonyl-hydrazide (TSH) was used as the foaming agent and 42.5% wt of phenolic resin was used as a binder. All the chemical reagents were homogenized in a conventional ball mill for 1 h. The powder technology and polymer foaming process utilized dry mixing of the abovementioned ceramic powders. The thermal cycle for forming the scaffolds was carried out in three stages: foaming, pyrolysis, and sintering. Foaming originates from melting the binder (~80°C), creating a suspension of phosphate-based bioactive glass particles and β-TCP. At this stage, the foaming agent decomposes and releases gas, expanding the structure and producing interconnected porosity on the scaffold.

Pyrolysis consists of decomposing the polymeric binder at a temperature of 500°C and sintering provides mechanical resistance to the scaffold by forming necks between particles. In a previous investigation by C. Ruiz-Aguilar et al. [20], the addition of the binder and foaming agent was analyzed in various chemical compositions of the scaffolds; the scaffolds were cooled inside the muffle furnace, with a cooling time of 10°C/min, until they reached room temperature and the samples were removed. They concluded that the best results of the chemical, structural, and physical characterization for applications in long bones (tibia, femur, and fibula) were those obtained by the V1, VZ0.5, and VZ1 samples. Therefore, the decision was made to resume the fabrication of the samples and the *in vitro* evaluation of artificial saliva in the present study, indicating that sample V1 corresponds to the scaffold composed of β-TCP/bioactive glass without added zirconia, whereas samples VZ0.5 and V1.0 are the scaffolds formed by β-TCP/bioactive glass, to which 0.5% and 1.0% mol of zirconia were added, respectively.

### 2.4 Physical, chemical, and structural characterization

The compressive strength test of the scaffolds before immersion in artificial saliva was determined using a Karl Frank model 81105 universal machine at room temperature. The samples were cylindrical specimens, measuring 1 cm in diameter and 2 cm in length. The cylindrical scaffolds were compressed between two metal plates, with a strain rate of 0.5 mm/min and a load cell application of 1 kN, using n = 5.

A Field Emission Scanning Electron Microscope (FE-SEM) JEOL-JSM IT300 was operated at 20 kV to analyze the morphology and pore size of the scaffolds. HA formed after the samples were immersed in artificial saliva. Energy Dispersive Spectroscopy (EDS) was used to promptly inquire about the chemical elements present on the surface of the samples, after the immersion in artificial saliva, and to analyze the Ca/P ratio formed in the scaffolds. Five measurements were made per composition.

### 2.5 *In vitro* evaluation

For the *in vitro* tests, small cylinders measuring 0.5 cm in height and 1 cm in diameter were made with a weight of 0.5 g; they were later submerged in 6 ml of artificial saliva (VIRDEN; Mexico, La Homa Rd Mission, Tx) at different times (days 7 and 14). VIRDEN artificial saliva, composed of a concentration of mineral salts (Na^+^, F, K^+^, Cl_2_^-^, Ca^2+^, PO_4_^2-^, Mg^2+^), was used. The artificial saliva was changed every third day in all samples.

### 2.6 Statistical analysis

The before and after *in vitro* analyses were performed in triplicate in all samples. Moreover, the t-test significance level was 0.05.

## 3. Results

### 3.1 Compressive strength of scaffolds

The compressive strength results of the V1, VZ0.5, and VZ1.0 scaffolds were 1.0, 0.6, and 0.9 MPa and compared with the trabecular bone, cortical bone, enamel, and dentin, as seen in Table 1.

**Table 1:** Compressive strength values of the V1, VZ0.5, and V1.0 scaffolds compared with the values of trabecular and cortical bone [20].

Sample	Compressive resistance (MPa)	Elastic module (MPa)	Reference
V1	1.0 ± 0.02	357± 0.32	
VZ0.50	0.6 ± 0.43	574 ±0.83	
VZ1.0	0.9 ± 0.54	434 ± 0.42	
Cortical	130–200	7000–30000	[21]
Trabecular	0.1–16	50–500	
**Human enamel**	62.2± 23.8	1338.2 ± 307.9	[22]
**Human dentin**	193.7± 30.6	1653.7± 277.9	[22]

In the present investigation, the values are closer to those of trabecular bone. However, the porosity, geometry [23], distribution, interconnectivity, and morphology of the pores [24], as well as the manufacturing method [25] and the sintering temperature of the scaffolds, are all aspects that are crucial to consider, given that they play a critical role in the mechanical properties of the scaffolds [26]. Adding the surface roughness of the scaffold directly affects the bioactivity response [27].

The scaffold porosity reported in a previous study was V1 50±0.33%, VZ0.5 47±0.42, and VZ1.0 53±0.43 [20].

### 3.2 *In vitro* evaluation of scaffolds

**Fig 1:**
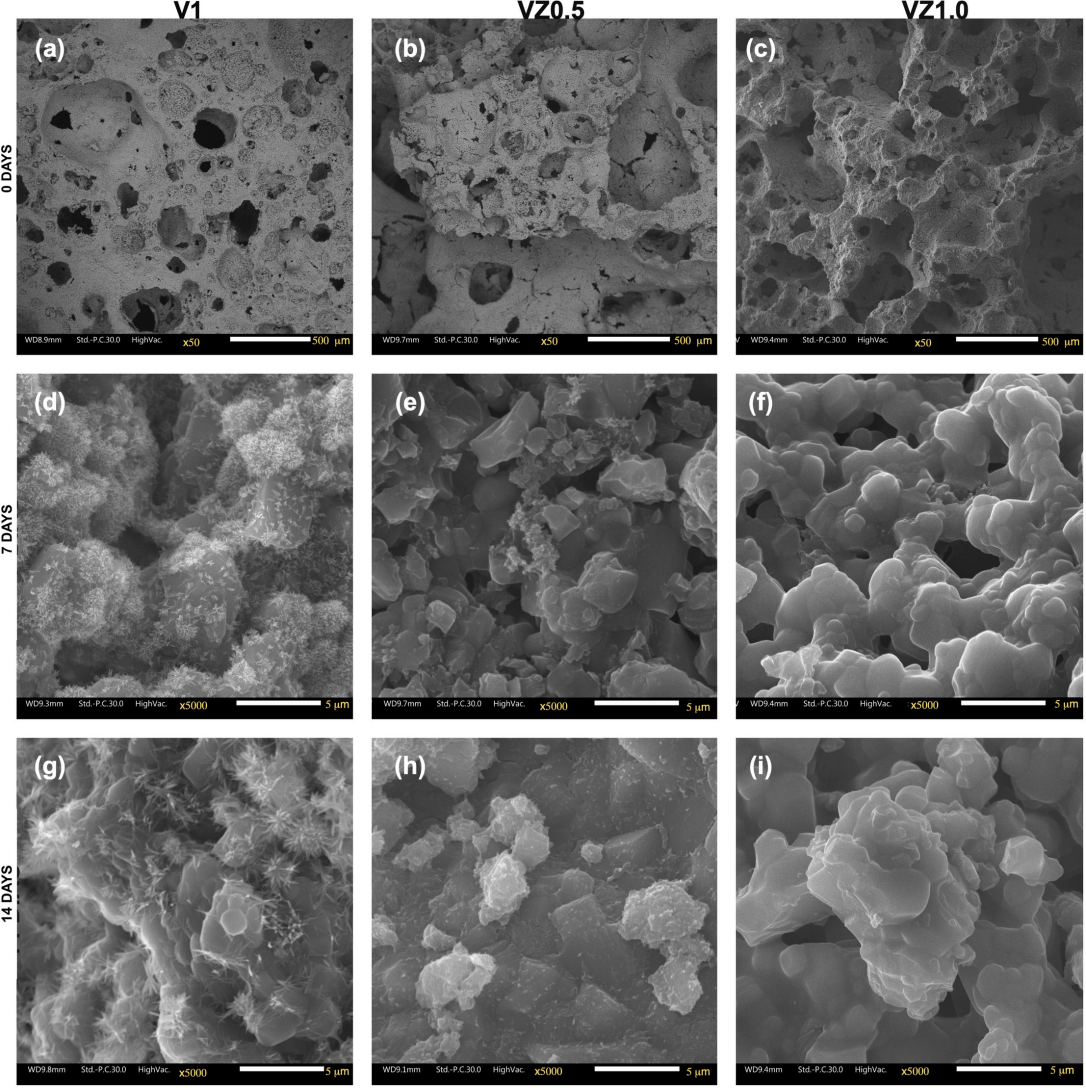
Scaffolds before being evaluated *in vitro* V1 (a), VZ0.5 (b) and VZ1.0 (d), after 7 days of immersion in artificial saliva V1 (d), VZ0.5 (e) and VZ1.0 (f), and after 14 days of immersion in artificial saliva V1 (g), VZ0.1 (h) and VZ0.1 (i).

The FE-SEM results showed the morphological changes of the scaffolds before and after being immersed in artificial saliva at days 7 and 14, as can be seen in Fig 1. Before being immersed in artificial saliva, the scaffolds presented a broad pore size distribution between 20 and 700 μm with circular morphology in the three compositions, V1, VZ0.5, and VZ1.0, as can be seen in Fig 1A–1C. The V1 scaffold from day 7 of immersion in artificial saliva presented significant morphological changes, the result of the interaction of scaffold ions and artificial saliva, forming the flower-like structure that covered a large part of the scaffold surface (Fig 1D), similar to that reported by other authors in previous investigations [28, 29]. The composition of VZ0.5 showed lower precipitation of the flower-like morphology, because of the 0.5 mol% zirconia content; this dopant element reduced the time of the dissolution process of the scaffold (Fig 1E). On the other hand, in the scaffold with 1.0 mol% ZrO_2_, a slight smoothing of the angles between the pores was observed (Fig 1F) after 7 days of immersion in artificial saliva, showing greater dissolution control of the scaffold. Sample V1, after 14 days of immersion in artificial saliva, exhibited an increase in the size of the flower-like structures and a greater superficial coating on the scaffold because of the ionic saturation of the artificial saliva, which led to precipitation of the HA on the surface (Fig 1G). The VZ0.5 sample presented an increase in the flower-like structure corresponding to hydroxyapatite, as can be seen in Fig 1H. The VZ1.0 scaffold, after 14 days of immersion in artificial saliva, revealed the formation of superficial irregular agglomerates (Fig 1).

**Fig 2:**
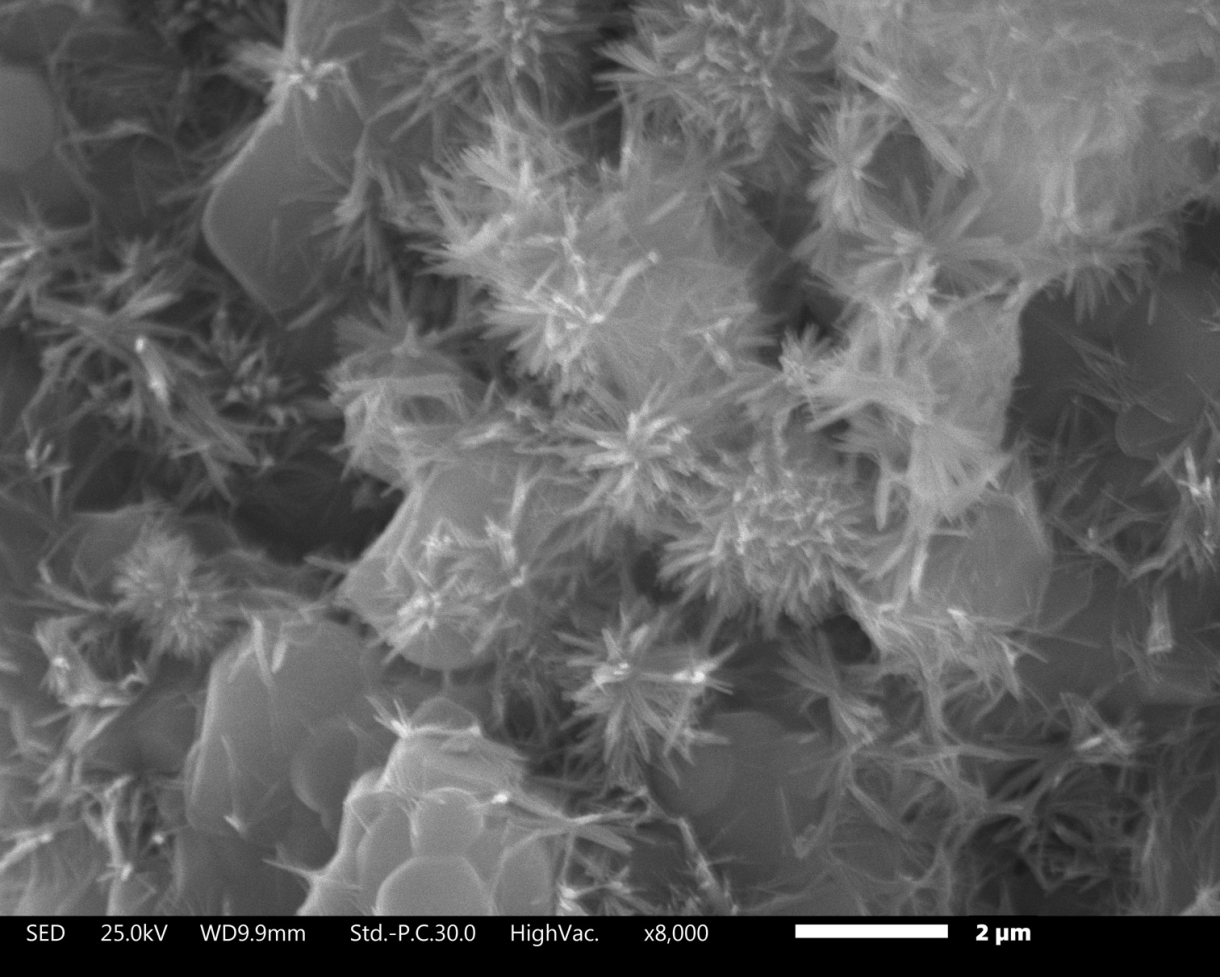
Flower-like structure of the V1 scaffold after 14 days of immersion in artificial saliva.

A physicochemical process formed the flower-like morphology in the V1 and VZ0.5 scaffolds. The supersaturation process resulted in the dissolution of Ca^2+^ and PO_4_^3-^ ions of the scaffolds and the interaction with saliva ions. The ions were preferentially grouped on the scaffold surface on the c-axis. They formed many hydroxyapatite rods, which grew radially, forming a chrysanthemum-like morphology (Fig 2), where each nucleus became the growth center of each flower, as reported by Xiaugou Liu et al. [30].

The dissolution phenomenon in the scaffolds generated calcium and phosphate ions that interacted with the artificial saliva. The formation of the hydroxyapatite phase was achieved by stabilizing the saliva at pH 7.4, as reported by Aiswarya Anil et al. [31]. The dissolution rate and biomineralization of the scaffolds had to maintain a constant chemical balance between the hydroxyapatite formed and the dissolution of the scaffolds. Importantly, other factors, such as microbial activity, the individual’s diet, and medium physicochemical properties, can alter the balance between the two phenomena described.

**Fig 3:**
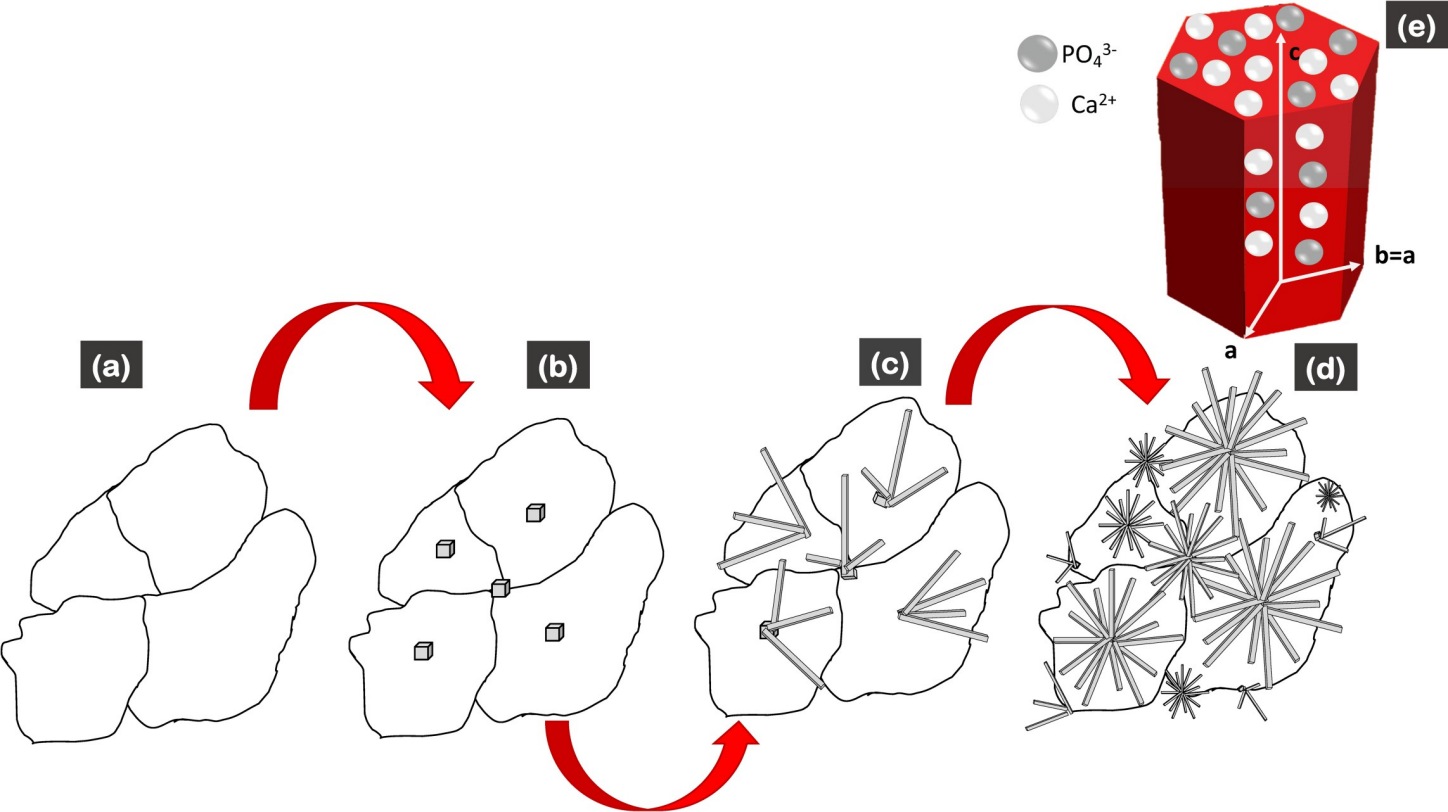
Representation of the formation process of hydroxyapatite crystals with flower-like morphology.

The formation process of the flower-like morphology of the scaffolds is represented in Fig 3A–3E. Before the scaffolds were immersed in artificial saliva, they showed a faceted morphology (Fig 3A). Subsequently, the dissolution process of the scaffolds in contact with artificial saliva generated the physicochemical phenomenon of ionic interaction of Ca^2+^ and PO_4_^3-^, allowing local supersaturation of calcium ions and favoring the nucleation of the hydroxyapatite crystals (Fig 3B).

The hexagonal structure HA crystal formed presented two surface areas with a more significant difference in electrical charges. The growth process of the previous HA crystalline phase continued, highlighting the fact that the HA hexagonal crystalline structures grow along the *c*-axis more efficiently, as it is a vital binding site for the Ca_9_(PO_4_)_6_ group in the [1] direction [20] (Fig 3C). Therefore, surface *c* is a predominant crystal growth facet, compared with surfaces *a* and *b*. The chrysanthemum-like morphology [32] originates due to many radially developed rods (Fig 3D); this phenomenon occurs because of the ionic interaction of the simulated medium (artificial saliva) with the two preferential surfaces on hexagonal HA crystals. The surface of *a* presents a greater affinity for cationic ions (Ca^2+^), whereas the surface of *c* has a greater tendency to accept anionic ions (PO_4_^3-^). However, the interaction of cationic and anionic ions can also occur, to a lesser extent, on the *c* and *b* axes [32], respectively, as shown in Fig 3E.

On the other hand, zirconium oxide showed a significant effect on the VZ0.5 and VZ1.0 scaffolds due to the superficial morphological changes observed in the samples; these morphological changes are attributed to the chemical phenomenon of breaking the oxide bonds of zirconium during the manufacturing method. The zirconium ion (Zr^4+)^ promoted the chemical bond with the calcium and pyrophosphate ions, forming two new crystalline phases after sintering, which were CaZrO_3_ and ZrP_2_O_7_. These results were analyzed and reported in two previous studies by C. Ruiz-Aguilar et al. [33]. These crystalline phases helped chemically stabilize the scaffold’s ionic solution, by controlling the release of Ca^2+^ and PO_4_^3-^ ions.

**Fig 4:**
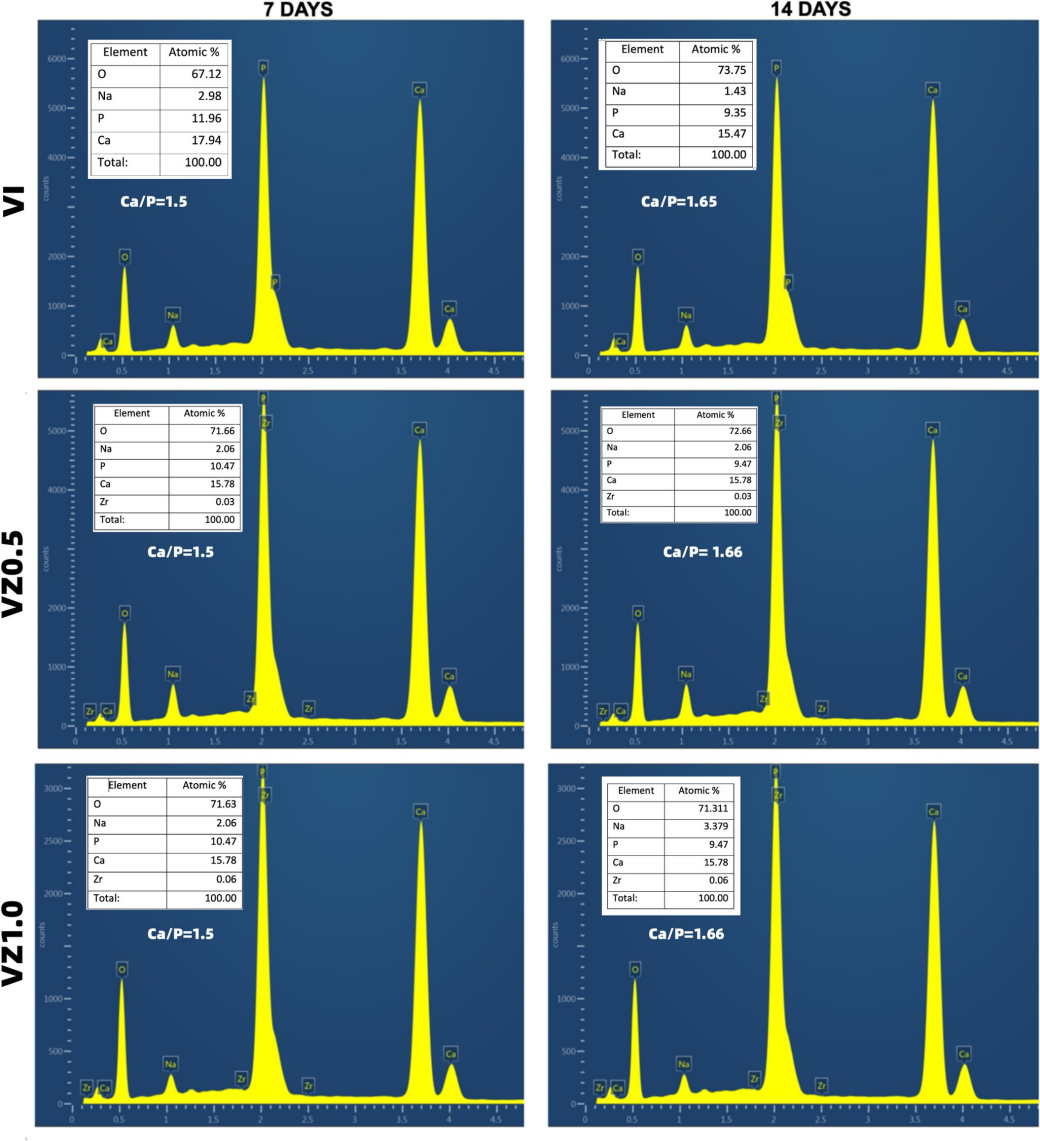
EDS images of the V1, VZ0.5, and VZ1.0 scaffolds, after 7 and 14 days of immersion in artificial saliva.

The EDS of the scaffolds showed the elements present in samples V1, VZ0.5, and V1.0 at days 7 and 14 after immersion in artificial saliva. The chemical elements of calcium and phosphorus were found in a more significant proportion in all the samples due to the chemical precursors used to manufacture the scaffolds in the present investigation. Subsequently, sodium, oxygen, and zirconium were found in less quantity, as seen in Fig 4.

**Fig 5:**
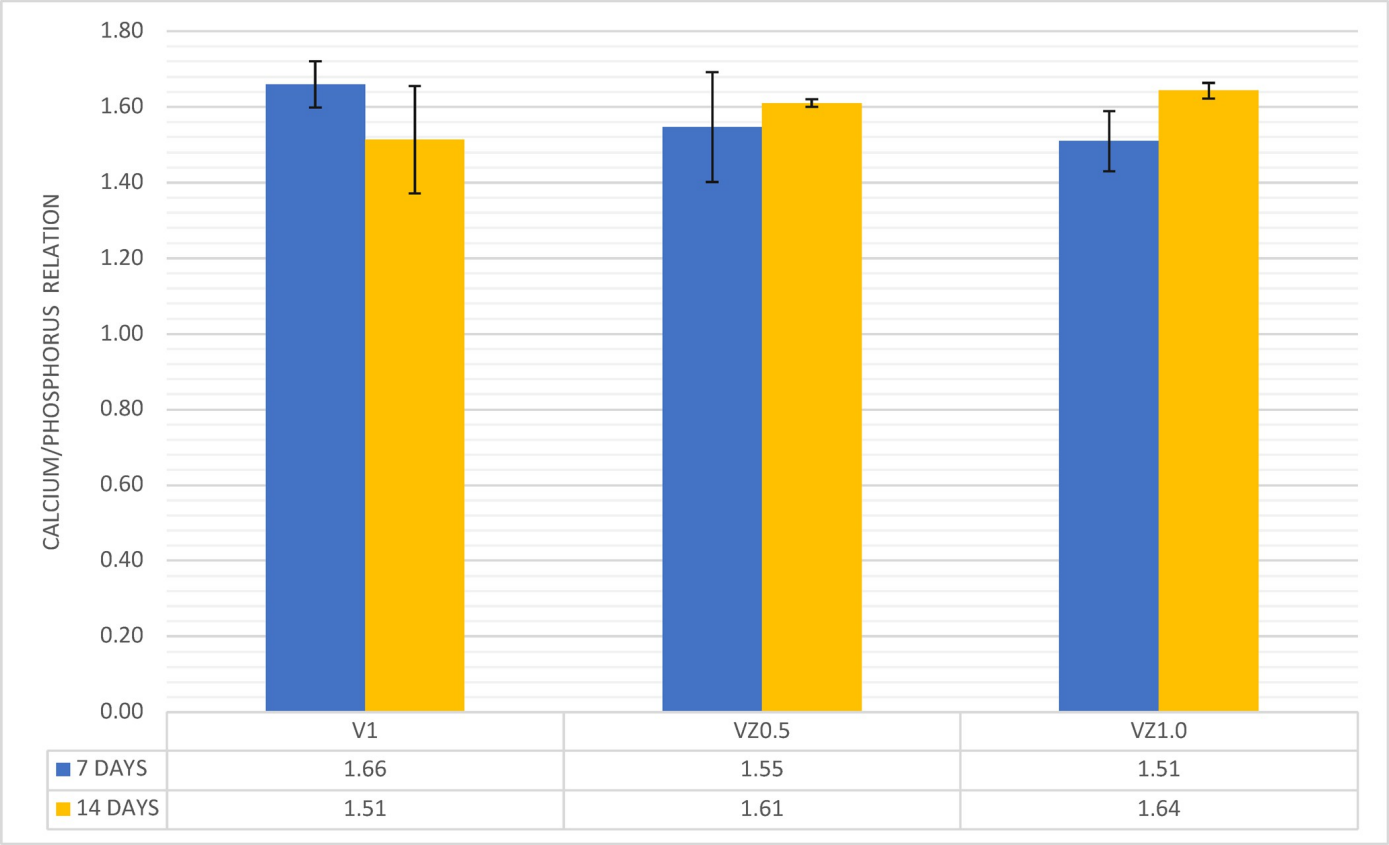
Ca/P ratio average of the V1, VZ0.5, and VZ1.0 scaffolds after immersion in artificial saliva at days 7 and 14, respectively.

Fig 5 shows the calcium and phosphorus ratios of the samples in the elemental atomic percentage obtained utilizing the EDS technique.

The values of the Ca/P ratio of the samples ranged from 1.5 to 1.66. These values allowed for predicting the dissolution behavior of the scaffolds, as well as the formation of HA after the immersion of the samples in artificial saliva at days 7 and 14. The value of the Ca/P = 1.5 ratio of the samples after 7 days of immersion in artificial saliva resulted from the lack of stoichiometry of the HA formed on the surface of the scaffolds—the reason why the ratio of calcium and phosphorus was less than 1.6. Subsequently, the crystalline phase of the HA was chemically balanced, obtaining the value of the Ca/P ratio = 1.6 in the VZ0.5 and VZ1.0 scaffolds; this value is identical to the theoretical value of the mineral phase of bone tissues (1.6667). The VZ0.5 and V1.0 samples, which were doped with ZrO_2_, presented the CaZrO_3_ (orthorhombic) [34] and ZrP_2_O_7_ (cubic) crystalline phases [35]. These two phases allowed better control of the VZ0.5 and VZ1.0 scaffolds to take place during the Ca^2+^ and PO_4_^3-^ ionic dissolution process—carrying out the resorption process of the ZrO_2_-doped scaffolds in a controlled manner and keeping the samples as temporary support structures for an adequate process of dental remineralization in more extended periods. In contrast, the V1 scaffold showed greater resorption, exhibiting significant dissolution effects after 7 days of immersion in artificial saliva, as seen in Fig 3.

Fluoride is the main ingredient in fluorinated products used for remineralization and prevention of caries progression. However, its use has a limitation: it reverses early lesions on the dental surface, leading to the remineralization of the porous superficial layer, generating the blockage of the enamel pores, and reducing the ionic exchange in the tooth. Enamel hinders the remineralization of the lesion and the complete remineralization of the tooth.

Fluoride-free remineralizing agents have proven to be an excellent alternative as gold standard medical treatment for early carious lesions on enamel. Tricalcium phosphate [36] stands out within this category, as does phosphate-based bioactive glass. The scaffold proposed in the present investigation shows the mass diffusion of calcium and phosphate in the lesion, enhancing the remineralization properties due to the interaction of saliva, without increasing the risk of dental calculus formation, and with a complete remineralization ratio. Additionally, titanium and titanium-based alloys are considered the benchmark for dental implant fixation. Nevertheless, they generate particles and titanium ions that accumulate in surrounding tissues due to the corrosion and wear of the metal, leading to bone loss caused by inflammatory reactions and dental osseointegration failure [37]. On the other hand, our results of the *in vitro* evaluation in artificial saliva showed that calcium is released, enabling its high availability on the surface of the lesion. Subsequent diffusion of calcium and phosphorus in the lesion will help remineralize dental lesions thanks to their excellent biocompatibility, promoting osteoconduction and osseointegration in the dental lesions due to the formation of HA, the mineral phase of the dental structure [14, 38].

Finally, it is essential to mention the powder metallurgy and polymer foaming techniques used to manufacture the scaffolds in the present investigation. These techniques gave the characteristics of the size and morphology of the pores, the chemical composition, mechanical properties, and the crystalline phases formed, before and after the *in vitro* evaluation [39] in artificial saliva 7 and 14 days after the scaffolds, which were aspects that directly influenced the results of this study, analyzing, and correlating the effect of zirconia on the bioactivity of scaffolds.

## 4. Conclusions

The V1, VZ0.5, and VZ1.0 scaffolds characterized physically, chemically, and *in vitro* using artificial saliva showed behavior like that of dental bone. Furthermore, zirconia was shown to help control the dissolution of the VZ0.5 and VZ1.0 scaffolds. However, sample V1 presented a more significant amount of surface precipitates with a chrysanthemum-like morphology corresponding to the crystalline phase of HA. This phase corresponds to the mineral structure of the teeth, and had preferential growth on the c-axis, forming random rods that grew from a nucleus formed due to the saturation of Ca^2+^ and PO_4_^3-^ ions. The results reported in the present investigation are promising.

Porous 3D phosphate-based bioactive glass /β-TCP scaffolds supply calcium and phosphate ions, by stimulating the formation of the mineral phase of enamel, through the release of calcium and phosphate ions from the scaffolds and the chemical and pH balance of simulated saliva, stimulating the formation of the mineral phase of dental structures.

Consequently, the VZ0.5 and VZ1.0 scaffolds can mimic natural tooth repair and thus be used as biomaterials for applications in the initial stages of caries remineralization, resulting in an alternative for the prevention and reduction of cavities in the Mexican population.

However, it is essential to complete *in vitro* and *in vivo* studies, ensuring that the scaffolds can be used for dental remineralization treatment.
